# Functional connectivity signatures of major depressive disorder: machine learning analysis of two multicenter neuroimaging studies

**DOI:** 10.1038/s41380-023-01977-5

**Published:** 2023-02-15

**Authors:** Selene Gallo, Ahmed El-Gazzar, Paul Zhutovsky, Rajat M. Thomas, Nooshin Javaheripour, Meng Li, Lucie Bartova, Deepti Bathula, Udo Dannlowski, Christopher Davey, Thomas Frodl, Ian Gotlib, Simone Grimm, Dominik Grotegerd, Tim Hahn, Paul J. Hamilton, Ben J. Harrison, Andreas Jansen, Tilo Kircher, Bernhard Meyer, Igor Nenadić, Sebastian Olbrich, Elisabeth Paul, Lukas Pezawas, Matthew D. Sacchet, Philipp Sämann, Gerd Wagner, Henrik Walter, Martin Walter, PsyMRI, Guido van Wingen

**Affiliations:** 1 Amsterdam UMC location University of Amsterdam, Department of Psychiatry, Meibergdreef 9, Amsterdam, The Netherlands; 2 Amsterdam Neuroscience, Amsterdam, The Netherlands; 3 Department Of Psychiatry and Psychotherapy, Jena University Hospital, Jena, Germany; 4 Department of Psychiatry and Psychotherapy, Medical University of Vienna, Vienna, Austria; 5 Indian Institute of Technology (IIT), Ropar, India; 6 Institute for Translational Psychiatry, University of Münster, Münster, Germany; 7 Department of Psychiatry, The University of Melbourne, Melbourne, VIC Australia; 8 Department of Psychiatry and Psychotherapy, Otto von Guericke University Magdeburg, Magdeburg, Germany; 9 German center for mental health, CIRC, Magdeburg, Germany; 10 Department of Psychology, Stanford University, Stanford, CA 94305 USA; 11 Department of Psychiatry, Charité Universitätsmedizin Berlin, Berlin, Germany; 12 Center for Social and Affective Neuroscience, Department of Biomedical and Clinical Sciences, Linköping University, Linköping, Sweden; 13 Department Of Psychiatry, University of Marburg, Marburg, Germany; 14 Department of Psychiatry, Psychotherapy and Psychosomatics, University Hospital of Zurich, Zurich, Switzerland; 15 Center for Depression, Anxiety, and Stress Research, McLean Hospital, Harvard Medical School, Belmont, MA USA; 16 Max Planck Institute of Psychiatry, Munich, Germany; 17 Charité-Universitätsmedizin Berlin, corporate member of Freie Universität Berlin and Humboldt-Universität zu Berlin, Department of Psychiatry and Psychotherapy, Charitéplatz 1, D-10117 Berlin, Germany

**Keywords:** Diagnostic markers, Depression

## Abstract

The promise of machine learning has fueled the hope for developing diagnostic tools for psychiatry. Initial studies showed high accuracy for the identification of major depressive disorder (MDD) with resting-state connectivity, but progress has been hampered by the absence of large datasets. Here we used regular machine learning and advanced deep learning algorithms to differentiate patients with MDD from healthy controls and identify neurophysiological signatures of depression in two of the largest resting-state datasets for MDD. We obtained resting-state functional magnetic resonance imaging data from the REST-meta-MDD (*N* = 2338) and PsyMRI (*N* = 1039) consortia. Classification of functional connectivity matrices was done using support vector machines (SVM) and graph convolutional neural networks (GCN), and performance was evaluated using 5-fold cross-validation. Features were visualized using GCN-Explainer, an ablation study and univariate t-testing. The results showed a mean classification accuracy of 61% for MDD versus controls. Mean accuracy for classifying (non-)medicated subgroups was 62%. Sex classification accuracy was substantially better across datasets (73–81%). Visualization of the results showed that classifications were driven by stronger thalamic connections in both datasets, while nearly all other connections were weaker with small univariate effect sizes. These results suggest that whole brain resting-state connectivity is a reliable though poor biomarker for MDD, presumably due to disease heterogeneity as further supported by the higher accuracy for sex classification using the same methods. Deep learning revealed thalamic hyperconnectivity as a prominent neurophysiological signature of depression in both multicenter studies, which may guide the development of biomarkers in future studies.

## Introduction

With more than 163 million people affected [1], major depressive disorder (MDD) is the most common psychiatric disorder in the world. This number keeps increasing every year, adding urgency to the question of how to diagnose, prevent, and treat it [2]. The promise of artificial intelligence for medicine also sparked the interest for using machine learning techniques for the development of biomarkers in psychiatry [3]. A meta-analysis of initial small-scale studies suggested that resting-state functional magnetic resonance imaging (fMRI) may provide highly accurate biomarkers for MDD [4]. However, neuroimaging biomarkers showed lower accuracies for other psychiatric disorders when based on large scale datasets, presumably due to increased heterogeneity within the patient group [5]. Until now, large scale resting-state cohorts for MDD have not been available, limiting the progress of the development of biomarkers for MDD.

In this work, we used data from two of the largest consortia (REST-meta-MDD (http://rfmri.org/REST-meta-MDD) [6] and PsyMRI (http://psymri.com), from now on *mddrest* and *psymri*) that obtained resting-state fMRI data across different research centers from patients with MDD and matched healthy controls (HC) to evaluate the potential of resting-state functional connectivity (FC) as biomarker for MDD.

FC between brain regions refers to the statistical dependence of neurophysiological signals [7], typically measured as Pearson correlation [8]. Until recently, the gold standard to explore brain differences was univariate-group-analysis, which interrogates one voxel at the time, and has revealed consistent FC differences in MDD [9]. However, univariate analysis potentially misses more complex patterns and is only able to detect average group differences. In the last few years, the increasing availability of machine‐learning (ML) and deep learning (DL) techniques [10] has enabled researchers to look into multivariate patterns. Recent results using the popular ML classifier support vector machine (SVM) obtained up to 95% classification accuracy in small datasets [11, 12]. DL algorithms are advanced ML techniques that learn abstract representation of the input data as an integral part of the training process. DL may have huge potential for high-dimensional data such as neuroimaging [13]. DL has shown convincing early results in many tasks involving image analysis, including classification of psychiatric disorders (see [14] for a review). Specific deep learning models on graphs (i.e., graph convolutional networks; GCN) have recently emerged, and demonstrated powerful performance on various tasks. Generally speaking, GCN models are a type of neural network architecture that can specifically leverage the graph structure that is typical for FC [15]. GCNs also enable the visualization of the important features to counter the typical criticism of ML for being “black-boxes” [16], and enable their use for uncovering the neural signatures of psychiatric disorders.

In the research reported here, we trained linear and nonlinear (rbf) SVM and spatial GCN classifiers on the *mddrest* (selected *N* = 2338) and *psymri* (selected *N* = 1039) datasets separately as well as combined. We performed two complimentary post-hoc visualization experiments: GCN-Explainer [17], which highlights the important connections between those brain regions that are necessary for the classifier to distinguish between MDD and controls; and an ablation study in which each brain region is systematically excluded (virtually ablated) one by one from the model. The consequent drop in accuracy from the original model accuracy indicates the contribution of the excluded region to the overall performance. To assess whether identified connections were stronger or weaker in MDD, we used group-level t-tests. Furthermore, as clinical heterogeneity is expected to have a large influence on the classification accuracy, we performed additional classifications for medicated and non-medicated patients separately.

## Methods and materials

### Datasets

The *psymri* consortium consists of 23 cohorts from across the world, including raw data from 531 patients (60% Males, 33.7 +/− 11.6 years old) and 508 controls (65% Males, 35.1 +/− 12.2 years old). The *mddrest* dataset collected byREST-meta-MDD Project is currently the largest resting-state fMRI database for MDD, including 1255 patients (57% Males, 36.6 +/− 15.7 years old) and 1083 HC (62% Males, 35.1 +/− 14.7 years old) from 25 cohorts in China. Supplementary Fig. S1 in the Supplementary Materials shows the distribution of participants between sites of the datasets. Demographic data are reported in Supplementary Table S1 for each of the classification tasks separately (see Supplementary Information for more details about sample composition).

We also utilized samples from two external rs-fMRI datasets that do not target MDD to benchmark classification performance on an independent task. *Abide* [18] is a comparable retrospective multicenter neuroimaging consortium but with patients with autism spectrum disorders (ASD) instead of MDD. In this study we used a sample of (*N* = 2000 (1590 M/410 F), 1030 ASD/970 TD) from both the first and second releases. The *UK Biobank* [19] is a prospective population cohort with harmonized data acquisition. We used a randomly sampled subset of the resting-state fMRI dataset with a comparable sample size to our MDD consortia (*N* = 2000, 1000 M/1000 F).

Anonymized data were made available for these consortia from studies that were approved by local Institutional Review Boards. All study participants provided written informed consent at their local institution.

### Data processing

**Fig. 1:**
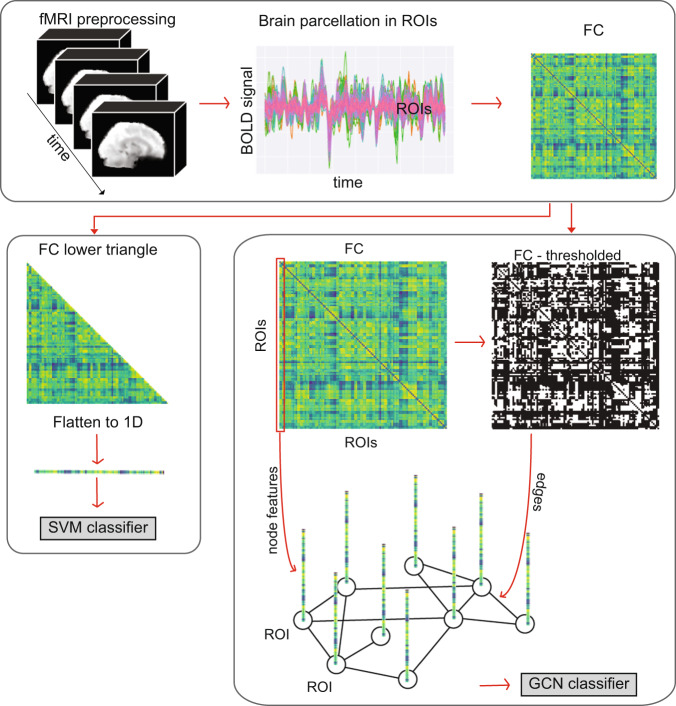
Pipeline from 4D rs-fMRI data to input for the classification task. Visual representation of our pipeline. For the *psymri* dataset, preprocessing of the raw 4D rs-fMRI and parcellation of the brain in regions of interest (ROIs) according to the Harvard-Oxford atlas was performed in house, while the *mddrest* consortium provided us directly with the time course of the same ROIs. The functional connectivity (FC) matrix was calculated using Pearson correlation between ROIs. Each entry in the FC represented the strength of functional connectivity between two ROIs, each row represented the correlation profile between one ROI and other ROIs. Since the FC is symmetrical, only one of the triangles was used as input for the SVM classifiers. From the FC we constructed the graph, which was used as GCN input. The ROIs were used as the nodes of the graphs. To construct the edges between nodes, i.e., the FC between ROIs, we first binarized the FC matrix so that only the 50th highest absolute values of the correlations of the matrix were transformed into ones, while the rest were transformed into zeros. We then drew an edge between ROIs whose correlation survived the binarization process. A feature was assigned to each node. The features were the original (i.e., before binarization) correlation profile of the node itself with the rest of the ROIs in the brain, therefore an entire row of the FC. SVM support vector machine, GCN graph convolutional network.

Standard preprocessing of the *psymri* dataset was done in house using FSL and ANTs (see Supplementary Information). Standard preprocessing of the *mddrest* data was done at each site using the Data Processing Assistant for Resting-State fMRI (DPARSF), which is based on SPM [20, 21] (see Supplementary Information for preprocessing of Abide and UK Biobank). Time courses of cortical and subcortical regions as defined by the Harvard-Oxford atlas [22] were extracted for all datasets (112 regions in total, see Supplementary Information for analyses on a functional atlas). Correlations between all brain regions were estimated and the resulting correlation matrices were used as features to predict class membership (Fig. 1). We used medication status to define more homogeneous groups, and included sex to benchmark classification performance for a task that is not dependent on the psychiatric diagnosis, resulting in the following classification tasks:

I. MDD vs HC
II. Non-medicated MDD vs HC
III. Medicated patients MDD vs HC
IV. Medicated MDD vs non-medicated MDD
V. Male vs female

For each contrast separately, we subsampled the classes so that the number of participants per class was equal. Supplementary Table S1 describes the sample compositions of the groups, for each contrast separately.

### Classifier models

Three classes of models, linear SVM, non-linear rbf SVM, and GCN, were used to evaluate prediction performance for all tests. To assess the generalizability of our results to data that have not been used to train the model, we used a 5-fold cross-validation (CV) scheme. Hyperparameter search for each model was based on best practices from the literature, and chosen empirically on the basis of a relative prediction accuracy on 20% of the training set [10] (See Supplementary Information for details). After the best hyperparameter combination had been determined, the actual performance of the classifiers was assessed on the test set [23]. The overall performance was calculated by averaging balanced accuracy performance in the five rounds on the test splits. Other evaluation metrics, namely F1-score, specificity and sensitivity, are reported in the Supplementary Information. All performances were compared against chance level using a random permutation test, then Bonferroni correction was used to adjust for the number of comparisons (Supplementary Information). Finally, for the contrast of MDD vs HC, to assess model generalization between datasets, we trained a model on one dataset and evaluated the model on the other dataset. For GCN we used a 5-fold cross-validation (CV) scheme to perform model selection on 20% of the test set. The SVMs do not need model selection and we applied a ‘one-shot’ procedure.

#### Linear and rbf SVM

**Fig. 2:**
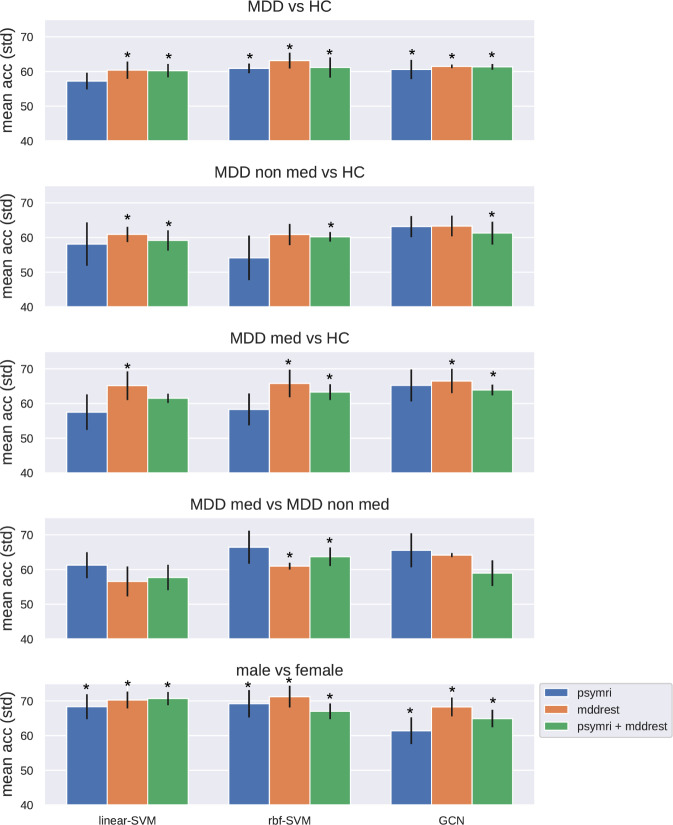
Performance of each classifier for each comparison, expressed as average balanced accuracy across five folds. Error bars indicate standard deviation across folds, * indicates classification results better than chance level after permutation testing. Significance level was corrected for the number of experiments performed, using the Bonferroni procedure.

We used linear and rbf SVM, popular classifiers that find respectively linear and non-linear combinations of features that best separate classes among the observations [24, 25].The upper triangular portion of the FC matrix was used as input for both linear and rbf SVM (Fig. 2).

#### GCN

Spatial GCN, referred to simply as GCN here, is a particular class of GCN. The first step consisted in transforming the FC matrices in graph representations. A graph representation is composed of nodes, nodal features or embeddings, and edges connecting the nodes. In our case, each node represents a region of interest (ROI). To construct the edges of the graphs, we thresholded the FC and binarized it so that the top 50% of connections in terms of connectivity strength were transformed into ones and the rest into zeros, regulating the sparsity of the graph. This threshold was derived from previous studies, including a systematic search for optimal graph sparsity by our own group. The nodal features were defined by the connectivity profile of that region to other regions, meaning the corresponding row in the FC matrix before thresholding (Fig. 1). This allowed the model to abstract information from each group of regions that have high temporal correlation. The GCN architecture used for this work was optimized for each contrast and dataset. See Supplementary Fig. S2 for a visual representation of the model and related concepts and Supplementary Information for details about the architecture. We used a binary cross entropy loss function and optimized the weights using Adams optimizer. The model is trained for 100 epochs with an initial learning rate of 0.001 decaying by a factor of 10 every 30 epochs.

### GCN-Explainer and ablation study

Two complementary experiments were carried out on the main MDD vs HC contrast. To assess the consistency of the results (e.g., replication), we performed the experiment on the *psymri* and the *mddrest* datasets independently. Regions highlighted by both datasets are reported in the results section. We focused on visualization of the GCN results because the methods allowed us to use complementary visualization techniques and strategies to enhance reliability of the results.

GCN-Explainer shows the manner in which the GCN classifier made the predictions. These explanations are in the form of a subgraph of the entire graph the GCN was trained on, so that the subgraph maximizes the mutual information with GCN prediction. This is achieved by formulating a mean field variational approximation and learning a real-valued graph mask which selects the important subgraph of the GCN’s computation graph.

We additionally performed an ablation study to identify the regions that influenced the performance of the GCN model in separating HC and MDD patients. This was done by masking the connectivity profile of each region, i.e., deleting the corresponding row from the connectivity matrix of the test set. The resultant drop in accuracy from the performance of the model trained on the full connectivity matrix is attributed to the region. We repeated the train-test process masking each region 10 times and calculated the mean drop in accuracy. The repetition leverages the stochastic nature of the GCN classifier to enhance the replicability of the results.

### Univariate group analyses

Univariate independent sample t-tests were performed on FC for the *psymri* and *mddrest* datasets separately. Sex, age, recording site and movement during scanning (average framewise displacement according to Jenkinson) were regressed out before testing. For each contrast and datasets, results were FDR corrected for multiple comparisons (*p* < 0.05).

## Results

### Classification performance

Classifications for the main comparison between MDD vs HC were significantly better than chance level after correction for multiple comparisons (with the exception of linear SVM classification of *psymri*), though balanced accuracies averaged across folds were low with a mean of 61% across datasets and classification models (range 57–63%; see Fig. 2 and Supplementary Table S2). Average balanced accuracies for the comparisons between medicated MDD vs HC, non-medicated MDD vs HC, and medicated MDD vs non-medicated MDD were comparable with a mean of 62% (range 54–67%). At least one classification model was significantly better than chance for each of these three comparisons for *mddrest* and the combination of *mddrest* + *psymri*, while none of the classifications for *psymri* were significant. The Supplementary Information provides additional evaluation metrics showing that sensitivity and specificity were balanced (Supplementary Tables S3–S5), and that site harmonization using Combat had little influence on the results (Supplementary Table S15). Comparable classification results were obtained when using a fully connected deep learning model or when using a functional instead of structural parcellation atlas (see Supplementary Information).

The cross-dataset training procedure for the contrast MDD vs HC resulted in lower performances. A GCN trained on *psymri* and tested on *mddrest* performed with a mean accuracy of 54.16 (sd = 0.66), while trained on the *mddrest* and tested on the *psymri* performed with mean accuracy of 56.38 (sd = 0.84), a SVM-linear performed with accuracy of 55.7 and 54.8 respectively on the same contrasts, and a SVM-rbf performed with accuracy of 53.1, and accuracy of 56.1.

To investigate the influence of subject and research site characteristics on classification performance, we assessed the accuracy for the different sexes, diagnostic statuses, scanner manufacturers and recording sites for the SVM-rbf that performed best. Particularly the variability in accuracy across sites was appreciable (range 48–87%), but was not significantly associated with sample size (r_s_ = 0.25, *p* = 0.25). Additional univariate t-testing revealed no significant FC difference between correctly and incorrectly classified participants (see Supplementary Information).

#### Symptom severity

To evaluate whether symptom severity could be predicted from the FC matrices, we used GCN and support vector regression (SVR) with the rbf kernel to predict Hamilton depression scores (HAM-D) for 1113 patients in *mddrest* and 333 patients in *psymri*. SVR could only explain 3.5-7% of the variance and GCN only predicted the training mean, indicating that symptom severity could not be predicted reliably.

### GCN-Explainer and ablation study results

**Fig. 3:**
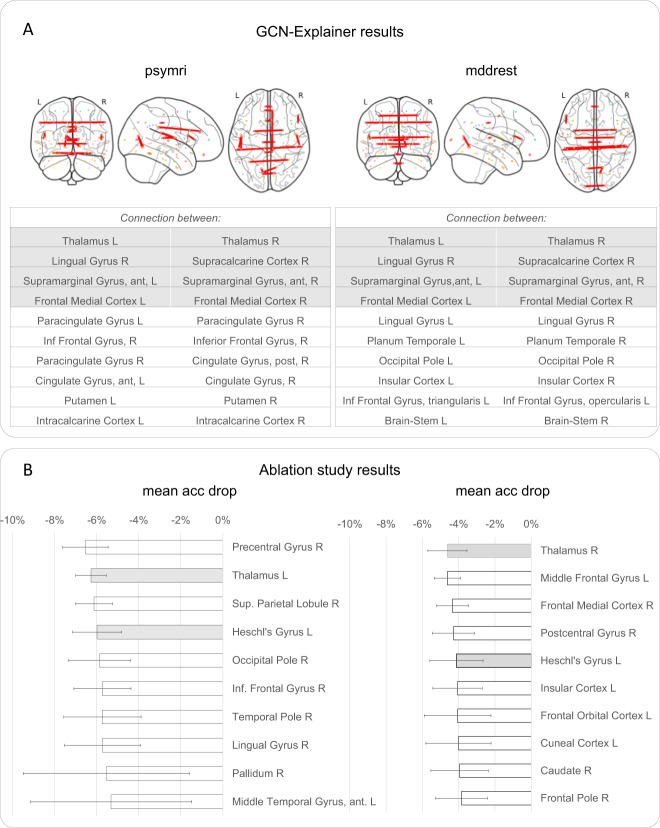
GCN explainer and ablation results for the classification of MDD and HC. **A** Results of the GCN explainer experiment obtained using the *psymri* dataset (left panel) and on the *mddrest* dataset (right panel): on top is the graphic representation of the functional connections between areas identified as necessary to discriminate MDD from HC, which are listed. The results on the left panel were obtained from the experiment on the *psymri* dataset, while those on the right are from the *mddrest* dataset. Connections identified by experiments in both datasets are shaded in gray. **B** Results of the ablation experiment obtained using the *psymri* dataset (left panel) and the *mddrest* dataset (right panel). Regions identified by experiments in both datasets are shaded in gray. L left, R right, ant anterior, inf. inferior, post posterior, acc balanced accuracy.

To gain insight into the most important connections for the classification of MDD vs HC, we performed two complementary visualization experiments on the *mddrest* and *psymri* datasets separately to assess whether results would be consistent. Visualization of the GCN using GCN-Explainer identified the connections between 1) the left and right thalamus, 2) the right lingual gyrus and right supracalcarine cortex, 3) the left and right anterior divisions of the supramarginal gyrus, and 4) left and right medial frontal cortex. These connections were amongst the ten most influential connections that were present in both datasets (Fig. 3A). An ablation study showed the highest drops in balanced accuracy that were present in both datasets for the thalamus (mean (sd) over 10 repetitions per fold; −6.27(2.17)% for *psymri*: and −4.62(1.08)% for *mddrest*:) and Heschl’s gyrus (−5.99(3.88)% for *psymri*, −4.12(1.47)% for *mddrest*). The results for *psymri* and *mddrest* are presented in Fig. 3B and Supplementary Table S6.

### Univariate group analyses

**Fig. 4:**
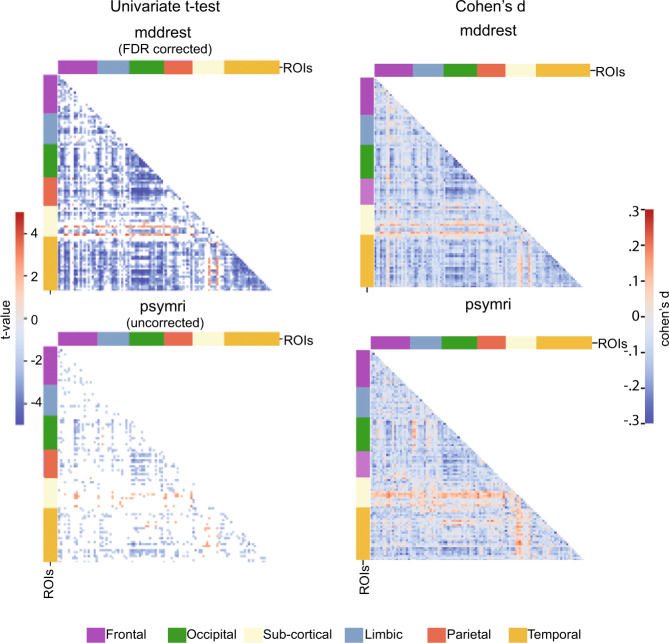
Univariate t-test results and Cohen’s *d*. Left: Results of the univariate t-test for the classification task MDD vs HC for the *mddrest* dataset (top) and for the psymri (bottom). The *mddrest* results are corrected for multiple comparison and thresholded using FDR < 0.05. The red lines correspond to the left and the right thalami. For the *psymri* dataset, t-tests did not survive correction for multiple comparisons, and the results are thresholded at *p*-uncorr < 0.05 to illustrate the comparable pattern as for *mddrest*. The clustering for lobes is done merely for illustration purposes. Right: Cohen’s *d* for the classification task MDD vs HC for the *mddrest* dataset (top) and for the *psymri* (bottom), calculated for each voxel group comparison.

28% of the connections showed significant differences between MDD and HC in the *mddrest* dataset, with predominantly reduced FC in MDD. For example, the amygdala showed reduced connectivity with 154 other regions, the insula showed reduced connectivity with 126 other regions, and the anterior cingulate cortex showed reduced connectivity with 100 other regions, but increased connectivity with the right precentral and postcentral gyri. In contrast, the thalamus showed *increased* connectivity with 199 other brain regions, primarily with frontal and insular regions, but decreased connectivity between interhemispheric homologs (Fig. 4). Effect sizes were low [26], with an average Cohen’s-*d* of −0.14 (range −0.34, −0.08) across significantly reduced connections, and an average Cohen’s-*d* of 0.12 (range 0.08, 0.18) for significantly increased thalamus connections.

In the *psymri* dataset, only the decreased connectivity between the left and right supracalcarine cortices survived correction for multiple comparisons for the comparison between MDD and HC. Inspection of the uncorrected results showed a comparable pattern of results as for the *mddrest* dataset (Fig. 4). Full results for each contrast and dataset (FDR corrected) are presented in Supplementary Tables S8–S13.

Only two comparisons, MDD vs HC and MDD-med vs HC showed replicable differences in the two datasets (Table 1). In the first contrast, connectivity between the left and right supracalcarine cortex showed a reduction in patients (*psymri* t: −4.73 *p*-corr < 0.05, *mddrest* t: −3.69, *p*-corr < 0.0005), out of the 3536 significant connectivity in the *mddrest* dataset. For the second comparison, connectivity between the left thalamus and left prefrontal gyrus showed increased connectivity in medicated patients (*psymri* t: 3.56 *p*-corr < 0.05, *mddrest* t: 3.03, *p*-corr < 0.05), while another nine FCs showed decreased FC in medicated patients, out of the 3527 significant connectivity in the *psymri* dataset and 596 in the *mddrest*.

**Table 1:** Replicated univariate t-test results in the *psymri* and *mddrest* datasets.

	*Connection between*		psymri		mddrest	
			*t*	*p*-value	*t*	*p*-value
MDD vs HC						
	R Supracalcarine Cortex	L Supracalcarine Cortex	−4.73	0.017	####	######
MDD med vs HC						
	L Thalamus	L Precentral Gyrus	3.56	0.048	3.03	0.034
	L Temporal Occipital Fusiform Cortex	L Lateral Occipital Cortex, infer.	−4.52	0.005	####	0.037
	R Supracalcarine Cortex	L Intracalcarine Cortex	−4.29	0.009	####	0.021
	R Superior Temporal Gyrus, pos.	R Superior Temporal Gyrus, ant.	−4.06	0.018	####	0.020
	R Planum Temporale	R Precentral Gyrus	−3.93	0.021	####	0.015
	L Frontal Orbital Cortex	R Frontal Medial Cortex	−3.75	0.032	####	0.049
	L Heschl’s Gyrus (includes H1 and H2)	R Postcentral Gyrus	−3.74	0.032	####	0.044
	R Supracalcarine Cortex	R Intracalcarine Cortex	−3.73	0.032	####	0.016
	R Putamen	R Middle Frontal Gyrus	−3.57	0.048	####	0.046
	L Central Opercular Cortex	R Precentral Gyrus	−3.56	0.048	####	0.012

Functional connectivity results showing differences between the MDD and HC group and the medicated MDD and the HC groups that are replicated in the two datasets. *T* values and *p*-values, FDR corrected, are reported separately for the psymri and mddrest dataset.

*R* Right, *L* Left.

### Sex classification

Classification of sex was beyond chance level for all the classifiers and datasets, with a mean across datasets and models of 68% (range 65–71%). To assess whether sex classification accuracy is comparable in other datasets, we performed similar analyses with comparable sample sizes (*N* = 2000) in the *Abide* and *UK Biobank* datasets. Sex classification accuracy was comparable in the retrospective *Abide* cohort (73%) and higher in the prospective harmonized *UK Biobank* cohort (81%).

## Discussion

The results showed that ML and DL classifiers were able to distinguish patients from controls beyond chance level, but that classification performance was low. Classification accuracies for (non-)medicated patients separately were comparable, suggesting that medication use had little influence on the results. Visualization of the functional connections that were most influential revealed hyperconnectivity of the thalamus. This was corroborated by two distinct visualization techniques and replicated in two datasets, suggesting that thalamic hyperconnectivity may be the most prominent neurophysiological characteristic of MDD. Interestingly, thalamic hyperconnectivity was rather specific, as MDD was mainly associated with widespread hypoconnectivity.

The 61% accuracy in these two datasets is considerably lower than the average 84% accuracy across small-scale studies in a recent meta-analysis [4]. Our results corroborate those from a recent Japanese multicenter study that reported a balanced accuracy of 67–69% [27]. The lower accuracy with larger sample sizes is paradoxical as ML and DL models only become better when trained on larger samples [28]. However, neuroimaging research has actually shown that prediction accuracy tends to decline with increasing sample size [29, 30]. This is presumably due to the increase in clinical heterogeneity when recruiting larger samples, as sample heterogeneity reduces model performance [5, 27]. We used data from two consortia that both consist of small samples obtained at many different research centers, and performances across sites ranged considerably (see Supplementary Information). Accordingly, the large total sample size came together with large heterogeneity, which is probably responsible for the poor accuracy of our model. Strategies to mitigate sites’ effect were not successful (see Supplementary Information). Heterogeneity is maximal when training and testing is performed on two different datasets, and indeed the lowest results obtained in the cross-datasets experiments confirmed the role of heterogeneity in compromising the final performance, which may be related to the distinct ancestry of the Chinese and European cohorts. Nevertheless, combination of both datasets led to comparable performance, indicating that it is possible to construct an MDD model that can generalize across cohorts. Though where sample homogeneity can lead to optimal model performance, sample heterogeneity ensures optimal generalization of the model to new data [5], suggesting that our results could form a lower bound on classification accuracy.

One way to reduce sample heterogeneity is to take clinical variability into account. We therefore split the sample into medicated and non-medicated patients. Although antidepressants are known to affect resting-state connectivity [31], this did not increase classification performance, suggesting that medication use had little influence on the classification results. Attempts to classify patients based on symptom severity or demographics were unsuccessful (see Supplementary Fig. S3). The diagnosis of MDD depends on the subjective evaluation of nine different symptoms and as little as one symptom may overlap between two patients [32], comorbidity is common, and symptoms may overlap with other disorders [33], leading to low interrater reliability of the diagnosis [34]. Such uncertainty associated with the diagnosis can obscure the relationship between a patient’s data and the category it belongs to [35–38], and thereby decrease accuracy [39]. Data-driven definition of the disorder and the use of biotypes could help arrive at more homogeneous psychiatric groups. The search for MDD-biotypes triggered a flurry of publications [40–45] and discussions in the last few years, but no consensus has emerged yet.

Another way to reduce heterogeneity of the dataset is to statistically harmonize data across centers. We performed data harmonization using Combat, which had little influence on the results. While this procedure can increase the power to detect group differences, it also had little influence on the classification of Alzheimer’s disease in the ADNI dataset [46]. While site harmonization will have a large effect on the ability to distinguish centers from the data, we expect that it will not have a large influence on the classification of interest when the dataset is balanced and site information is independent of group membership.

Despite our hypothesis that exploiting the graph-like structure of FC would be beneficial for the classification tasks, we found no clear advantage in using GCN over SVM, nor by another tested DL model (Supplementary Information). DL methods like GCNs perform especially well when applied to very large samples such as the 14 million images in ImageNet [47]. However, in applications like ours, the numerosity of the dataset is limited. Even this largest sample of MDD fMRI data might simply not be enough to exploit the potential of GCNs [48].

Although the results did not meet the accuracy criteria for a clinical diagnostic tool, the insights they provide go beyond “mere” prediction, and can help connect neurobiological processes to their psychiatric consequences (but see ref. [49]). In the classification between MDD and HC, the thalamus stood out as the only region whose importance is supported both by two different visualization techniques and the replication across two datasets. The GCN-Explainer identified the inter-hemispheric connections between left and right thalamus as most important to the classification. Of note, the prominent inter-hemispheric connections in the results (Fig. 3A) may reflect the nature of the way we constructed the GCN-layers (Supplementary Information). We therefore assume that the entire FC profile of the thalamus is driving the result, rather than only the interhemispheric connectivity. This interpretation is supported by the ablation study performed for the GCN for which we removed each thalamus and it’s FC with the rest of the brain and witnessed a ~5% drop in accuracy, confirming its role in discriminating between MDD and HC. Importantly, the relatively low accuracy of the classifiers influences the reliability of visualization techniques. For the GCN model, we were able to take advantage of the stochastic nature of the algorithm to increase the replicability of the results by repeating the training-test procedure 10 times. SVM algorithms are deterministic and this augmentation is not possible, limiting reliability of the results even further (reported in the Supplementary Information)

Additional univariate t-testing showed thalamic hyperconnectivity in *mddrest*, while most other brain regions showed hypoconnectivity. This pattern was also observed in *psymri*, but this did not withstand correction for multiple comparisons. Univariate effect sizes in the two datasets were comparable, suggesting that the *psymri* results could have been penalized by the smaller sample size. In general, the univariate effect sizes were negligible to small [26]. This highlights the usefulness of multivariate analysis, as the obtained ~60% accuracy translates into a medium effect size [50].

Initial studies [51, 52] as well as recent meta-analyses [53–57] have already pointed to thalamic hyperactivity, during rest as well as during cognitive and emotion processing. Other studies have suggested metabolic abnormalities in the thalamus of patients with depression [58–60], and specifically the mediodorsal thalamus was implicated in onset of depression [61, 62]. This nuclei is responsible for integrating sensory, motor, visceral and olfactory information and subsequently relating it to the individual’s emotional state [63] and its connectivity profile is congruent with the increased connectivity pattern we find in our study. This suggests that our results may be driven by hyperconnectivity of the mediodorsal thalamus. Hypervigilant brain states in MDD have been observed with electroencephalography [64, 65] and inversely, thalamic deactivation precedes sleep onset. EEG-fMRI-studies in healthy subjects have reported a thalamic BOLD signal decrease in lower vigilance states [64] and – directly relevant to our result – thalamocortical uncoupling as a general hallmark of (light) sleep [66–68]. Thalamic hyperconnectivity during MRI scanning (that represents a mild stress experiment) may well hint towards a general MDD-related dysfunction within the larger brain network, as recent views on the thalamus hold that it is not a passive relay station but that it has a central role in ongoing cortical functioning [69, 70]. Overall, this suggests a hypothesis that corticothalamic hyperconnectivity may ‘hijack’ the corticocortical connectivity that was reduced throughout the brain in our study.

This study has to be considered in light of its strengths and limitations. Its main strength is the use of two of the largest resting-state fMRI consortia with clinically confirmed MDD that show converging evidence for poor discrimination of MDD and the importance of thalamic hyperconnectivity. At the same time, these large datasets come with the limitation of large clinical (e.g., differences in severity and chronicity) and technological (e.g., differences in scanners and MRI acquisitions) heterogeneity, presumably reducing classification accuracy. To evaluate whether technolgical heterogeneity could have influenced the results, we compared sex classification in our MDD cohorts with sex classification in a comparable cohort for ASD (ABIDE) and a high quality dataset with prospective data acquisition harmonization (UK Biobank). The results show that sex classification accuracy can increase from (71–73%) in the MDD and ASD datasets to 81% in the UK Biobank. This suggests that our MDD classification result is as good as can be obtained from heterogenous retrospective multicenter cohorts. Though the higher sex classification accuracy in the UK Biobank suggests that the accuracy for MDD may improve when all data could be collected on the same scanner. A further limitation is that we analyzed FC as a stationary feature even though it consists of dynamic changes in neural activity over time, which may be important for the classification of MDD [71]. And finally, given the domain heterogeneity of psychiatric disorders, classifying a disease based on one brain imaging modality only is reductive. An integrative modeling of multimodal data, such as molecular, genomic, clinical, medical imaging, physiological signal and behavioral means comprehensively considering different aspects of the disease, thus likely enhancing the classification performance [13, 72]

In conclusion, our study provides a realistic and possibly lower bound estimate of the classification performance that can be obtained with the application of FC on a large, ecologically valid, multi-site sample of MDD patients. Our findings show that FC can distinguish between MDD patients and HCs, but that it is not sufficiently accurate for clinical use. Despite the low accuracy, visualization of the DL classifier enabled important insights into the neural basis of MDD, and revealed consistent and reproducible thalamic hyperconnectivity as the most prominent neurophysiological characteristic of MDD.

### Supplementary information

Supplementary_information

Table S1

Table S2

Table S3

Table S4

Table S5

Table S6

Table S7

Table S8

Table S9

Table S10

Table S11

Table S12

Table S13

Table S14

Table S15

Figure S1

Figure S2

Figure S3

## Data Availability

Deidentified and anonymized data were contributed from studies approved by local Institutional Review Boards. All study participants provided written informed consent at their local institution. Data of the PsyMRI project are available at http://psymri.org/. Data of the REST-meta-MDD project are available at: http://rfmri.org/REST-meta-MDD. Data that were generated and the graph convolutional models used for this study are available on request to the corresponding author.

## References

[1] GBD 2017 Disease and Injury Incidence and Prevalence Collaborators. (2018). Global, regional, and national incidence, prevalence, and years lived with disability for 354 diseases and injuries for 195 countries and territories, 1990-2017: a systematic analysis for the Global Burden of Disease Study 2017. Lancet.

[2] Coleman JRI, Gaspar HA, Bryois J, Breen G, Bipolar Disorder Working Group of the Psychiatric Genomics Consortium, Major Depressive Disorder Working Group of the Psychiatric Genomics Consortium (2020). The genetics of the mood disorder spectrum: genome-wide association analyses of more than 185,000 cases and 439,000 controls. Biol Psychiatry.

[3] Topol EJ (2019). High-performance medicine: the convergence of human and artificial intelligence. Nat Med.

[4] Kambeitz J, Cabral C, Sacchet MD, Gotlib IH, Zahn R, Serpa MH (2017). Detecting neuroimaging biomarkers for depression: a meta-analysis of multivariate pattern recognition studies. Biol Psychiatry.

[5] Schnack HG, Kahn RS (2016). Detecting neuroimaging biomarkers for psychiatric disorders: sample size matters. Front Psychiatry.

[6] Yan C-G, Chen X, Li L, Castellanos FX, Bai T-J, Bo Q-J (2019). Reduced default mode network functional connectivity in patients with recurrent major depressive disorder. Proc Natl Acad Sci USA.

[7] Friston KJ, Frith CD, Liddle PF, Frackowiak RS (1993). Functional connectivity: the principal-component analysis of large (PET) data sets. J Cereb Blood Flow Metab.

[8] Murrough JW, Abdallah CG, Anticevic A, Collins KA, Geha P, Averill LA (2016). Reduced global functional connectivity of the medial prefrontal cortex in major depressive disorder. Hum Brain Mapp.

[9] Hamilton JP, Etkin A, Furman DJ, Lemus MG, Johnson RF, Gotlib IH (2012). Functional neuroimaging of major depressive disorder: a meta-analysis and new integration of base line activation and neural response data. Am J Psychiatry.

[10] LeCun Y, Bengio Y, Hinton G. Deep learning. Nature. 2015;521:436–44. 10.1038/nature14539 26017442

[11] Zeng L-L, Shen H, Liu L, Wang L, Li B, Fang P (2012). Identifying major depression using whole-brain functional connectivity: a multivariate pattern analysis. Brain..

[12] Wang X, Ren Y, Zhang W (2017). Depression disorder classification of fMRI data using sparse low-rank functional brain network and graph-based features. Comput Math Methods Med.

[13] Durstewitz D, Koppe G, Meyer-Lindenberg A (2019). Deep neural networks in psychiatry. Mol Psychiatry.

[14] Quaak M, van de Mortel L, Thomas RM, van Wingen G (2021). Deep learning applications for the classification of psychiatric disorders using neuroimaging data: Systematic review and meta-analysis. NeuroImage Clin.

[15] Thomas NK, Welling M. Semi-supervised classification with graph con- volutional networks. arXiv. 2016. https://arxiv.org/abs/1609.02907.

[16] Castelvecchi D (2016). Can we open the black box of AI?. Nature.

[17] Ying R, Bourgeois D, You J, Zitnik M, Leskovec J (2019). GNNExplainer: generating explanations for graph neural networks. Adv Neural Inf Process Syst.

[18] Di Martino A, Yan CG, Li Q, Denio E, Castellanos FX, Alaerts K (2014). The autism brain imaging data exchange: towards a large-scale evaluation of the intrinsic brain architecture in autism. Mol Psychiatry.

[19] Sudlow C, Gallacher J, Allen N, Beral V, Burton P, Danesh J (2015). UK biobank: an open access resource for identifying the causes of a wide range of complex diseases of middle and old age. PLoS Med.

[20] Chao-Gan Y, Yu-Feng Z (2010). DPARSF: a MATLAB toolbox for “pipeline” data analysis of resting-state fMRI. Front Syst Neurosci.

[21] Penny WD, Friston KJ, Ashburner JT, Kiebel SJ, Nichols TE, editors. Statistical parametric mapping: the analysis of functional brain images. Elsevier; 2011.

[22] Makris N, Goldstein JM, Kennedy D, Hodge SM, Caviness VS, Faraone SV (2006). Decreased volume of left and total anterior insular lobule in schizophrenia. Schizophr Res.

[23] Hastie T, Tibshirani R, Friedman JH, Friedman JH. The elements of statistical learning: data mining, inference, and prediction. Vol. 2. New York: Springer; 2009. pp. 1–758.

[24] Fisher RA (1936). The use of multiple measurements in taxonomic problems. Ann Eugen.

[25] McLachlan GJ. Discriminant analysis and statistical pattern recognition. In: Wiley Series in Probability and Statistics. 1992. 10.1002/0471725293.

[26] Cohen J. Statistical power analysis. Curr Dir Psychol Sci. 1992;1:98–101.

[27] Yamashita A, Sakai Y, Yamada T, Yahata N, Kunimatsu A, Okada N (2020). Generalizable brain network markers of major depressive disorder across multiple imaging sites. PLoS Biol.

[28] Schnack HG, Nieuwenhuis M, van Haren NEM, Abramovic L, Scheewe TW, Brouwer RM (2014). Can structural MRI aid in clinical classification? A machine learning study in two independent samples of patients with schizophrenia, bipolar disorder and healthy subjects. Neuroimage..

[29] Wolfers T, Buitelaar JK, Beckmann CF, Franke B, Marquand AF (2015). From estimating activation locality to predicting disorder: a review of pattern recognition for neuroimaging-based psychiatric diagnostics. Neurosci Biobehav Rev.

[30] Bruin WB, Taylor L, Thomas RM, Shock JP, Zhutovsky P, Abe Y (2020). Structural neuroimaging biomarkers for obsessive-compulsive disorder in the ENIGMA-OCD consortium: medication matters. Transl Psychiatry.

[31] van Wingen GA, Tendolkar I, Urner M, van Marle HJ, Denys D, Verkes R-J (2014). Short-term antidepressant administration reduces default mode and task-positive network connectivity in healthy individuals during rest. Neuroimage..

[32] American Psychiatric Association. Diagnostic and statistical manual of mental disorders (DSM-5®). Washington, DC: American Psychiatric Pub; 2013. pp. 991.

[33] Insel T, Cuthbert B, Garvey M, Heinssen R, Pine DS, Quinn K (2010). Research Domain Criteria (RDoC): toward a new classification framework for research on mental disorders. Am J Psychiatry.

[34] Regier DA, Narrow WE, Clarke DE, Kraemer HC, Kuramoto SJ, Kuhl EA (2013). DSM-5 field trials in the United States and Canada, Part II: test-retest reliability of selected categorical diagnoses. Am J Psychiatry.

[35] Hickey RJ (1996). Noise modelling and evaluating learning from examples. Artif Intell.

[36] Nigam N, Dutta T, Gupta HP. Impact of noisy labels in learning techniques: a survey. Adv Data Inf Sci. 2020;403–11. 10.1007/978-981-15-0694-9_38.

[37] AbuDahab K, Xu D-L, Keane J. Induction of belief decision trees from data. In: AIP Conference Proceedings. 2012. 10.1063/1.4756644.

[38] Lim C, Han S, Lee J. Analyzing deep neural networks with noisy labels. In: 2020 IEEE International Conference on Big Data and Smart Computing (BigComp). 2020. 10.1109/bigcomp48618.2020.00012.

[39] Frénay B, Verleysen M (2014). Classification in the presence of label noise: a survey. IEEE Trans Neural Netw Learn Syst.

[40] Drysdale AT, Grosenick L, Downar J, Dunlop K, Mansouri F, Meng Y (2017). Resting-state connectivity biomarkers define neurophysiological subtypes of depression. Nat Med.

[41] Dinga R, Schmaal L, Penninx BWJH, van Tol MJ, Veltman DJ, van Velzen L (2019). Evaluating the evidence for biotypes of depression: methodological replication and extension of. Neuroimage Clin.

[42] Clementz BA, Sweeney JA, Hamm JP, Ivleva EI, Ethridge LE, Pearlson GD (2018). Identification of distinct psychosis biotypes using brain-based biomarkers. Focus..

[43] Grosenick L, Shi TC, Gunning FM, Dubin MJ, Downar J, Liston C (2019). Functional and optogenetic approaches to discovering stable subtype-specific circuit mechanisms in depression. Biol Psychiatry Cogn Neurosci Neuroimaging.

[44] Mihalik A, Ferreira FS, Moutoussis M, Ziegler G, Adams RA, Rosa MJ (2020). Multiple holdouts with stability: improving the generalizability of machine learning analyses of brain–behavior relationships. Biol Psychiatry.

[45] Ing A, Sämann PG, Chu C, Tay N, Biondo F, Robert G (2019). Identification of neurobehavioural symptom groups based on shared brain mechanisms. Nat Hum Behav.

[46] Chen AA, Beer JC, Tustison NJ, Cook PA, Shinohara RT, Shou H, Alzheimer’s Disease Neuroimaging Initiative. (2022). Mitigating site effects in covariance for machine learning in neuroimaging data. Hum Brain Mapp.

[47] Russakovsky O, Deng J, Su H, Krause J, Satheesh S, Ma S (2015). ImageNet large scale visual recognition challenge. Int J Comp Vis.

[48] He T, Kong R, Holmes AJ, Sabuncu MR, Eickhoff SB, Bzdok D, et al. Is deep learning better than kernel regression for functional connectivity prediction of fluid intelligence? In: 2018 International Workshop on Pattern Recognition in Neuroimaging (PRNI). 2018. 10.1109/prni.2018.8423958.

[49] Rudin C (2019). Stop explaining black box machine learning models for high stakes decisions and use interpretable models instead. Nat Mach Intell.

[50] Chinn S (2000). A simple method for converting an odds ratio to effect size for use in meta-analysis. Stat Med.

[51] Drevets WC, Videen TO, Price JL, Preskorn SH, Carmichael ST, Raichle ME (1992). A functional anatomical study of unipolar depression. J Neurosci.

[52] Greicius MD, Flores BH, Menon V, Glover GH, Solvason HB, Kenna H (2007). Resting-state functional connectivity in major depression: abnormally increased contributions from subgenual cingulate cortex and thalamus. Biol Psychiatry.

[53] Hamilton JP, Farmer M, Fogelman P, Gotlib IH (2015). Depressive rumination, the default-mode network, and the dark matter of clinical neuroscience. Biol Psychiatry.

[54] Palmer SM, Crewther SG, Carey LM, START Project Team. (2014). A meta-analysis of changes in brain activity in clinical depression. Front Hum Neurosci.

[55] Müller VI, Cieslik EC, Serbanescu I, Laird AR, Fox PT, Eickhoff SB (2017). Altered brain activity in unipolar depression revisited: meta-analyses of neuroimaging studies. JAMA Psychiatry.

[56] Mayberg HS (1997). Limbic-cortical dysregulation: a proposed model of depression. J Neuropsychiatry Clin Neurosci.

[57] Phillips ML, Drevets WC, Rauch SL, Lane R (2003). Neurobiology of emotion perception II: Implications for major psychiatric disorders. Biol Psychiatry.

[58] Holthoff VA, Beuthien-Baumann B, Zündorf G, Triemer A, Lüdecke S, Winiecki P (2004). Changes in brain metabolism associated with remission in unipolar major depression. Acta Psychiatr Scand.

[59] Dougherty DD, Weiss AP, Cosgrove GR, Alpert NM, Cassem EH, Nierenberg AA (2003). Cerebral metabolic correlates as potential predictors of response to anterior cingulotomy for treatment of major depression. J Neurosurg.

[60] Neumeister A, Nugent AC, Waldeck T, Geraci M, Schwarz M, Bonne O (2004). Neural and behavioral responses to tryptophan depletion in unmedicatedpatients with remitted major depressive disorder and controls. Arch Gen Psychiatry.

[61] Li W, Liu J, Skidmore F, Liu Y, Tian J, Li K (2010). White matter microstructure changes in the thalamus in Parkinson disease with depression: a diffusion tensor MR imaging study. AJNR Am J Neuroradiol.

[62] Young KA, Holcomb LA, Yazdani U, Hicks PB, German DC (2004). Elevated neuron number in the limbic thalamus in major depression. Am J Psychiatry.

[63] Price JL, Drevets WC (2009). Neurocircuitry of mood disorders. Neuropsychopharmacology..

[64] Olbrich S, Mulert C, Karch S, Trenner M, Leicht G, Pogarell O (2009). EEG-vigilance and BOLD effect during simultaneous EEG/fMRI measurement. Neuroimage..

[65] Hegerl U, Wilk K, Olbrich S, Schoenknecht P, Sander C (2012). Hyperstable regulation of vigilance in patients with major depressive disorder. World J Biol Psychiatry.

[66] Spoormaker VI, Sturm A, Andrade K, Schroeter M, Goya-Maldonado R, Holsboer F (2010). The neural correlates and temporal sequence of the relationship between shock exposure, disturbed sleep and impaired consolidation of fear extinction. J Psychiatr Res.

[67] Sämann PG, Wehrle R, Hoehn D, Spoormaker VI, Peters H, Tully C (2011). Development of the brain’s default mode network from wakefulness to slow wave sleep. Cereb Cortex.

[68] Tagliazucchi E, Laufs H (2014). Decoding wakefulness levels from typical fMRI resting-state data reveals reliable drifts between wakefulness and sleep. Neuron.

[69] Sherman SM (2016). Thalamus plays a central role in ongoing cortical functioning. Nat Neurosci.

[70] Weis S, Patil KR, Hoffstaedter F, Nostro A, Yeo BTT, Eickhoff SB (2020). Sex classification by resting state brain connectivity. Cereb Cortex.

[71] Yao D, Sui J, Yang E, Yap P-T, Shen D, Liu M. Temporal-adaptive graph convolutional network for automated identification of major depressive disorder using resting-state fMRI. Mach Learn Med Imaging. 2020. 1–10. 10.1007/978-3-030-59861-7_1. 10.1007/978-3-030-59861-7_1 PMC9645786 36383497

[72] Yang J, Yin Y, Zhang Z, Long J, Dong J, Zhang Y (2018). Predictive brain networks for major depression in a semi-multimodal fusion hierarchical feature reduction framework. Neurosci Lett.

