# Supplementary material for: Functional connectivity signatures of major depressive disorder: machine learning analysis of two multicenter neuroimaging studies
Source: Mol Psychiatry. 2023 Feb 15;28(7):3013–22. doi: 10.1038/s41380-023-01977-5 (PMC10615764; doi:10.1038/s41380-023-01977-5)
Supplement: Supplementary file 1 — Supplementary_information [file 41380_2023_1977_MOESM1_ESM.docx]

**Supplementary Information**

Datasets: sample composition and preprocessing

*Psymri*

The *psymri* dataset, collected by the PsyMRI consortium, is a collection of existing rs-fMRI data collected by 23 research groups, with a focus on major depressive disorder (MDD) and neurodegenerative diseases.

On average, each site contributed 31.2.0 ± 22.4 patients with MDD (range 10 to 93) and 29.9 ± 18.2 HC (range 2 to 69). Sites used different versions of the HAMD scale to diagnose MDD, so it is not possible to report a unified average of MDD severity. Most MDD patients were female (349 vs. 182 males).

What the consortium made available was raw functional data. The preprocessing of the PsyMRI data was done in house using the FSL FEAT [(1–4)](https://paperpile.com/c/fzIBvJ/iSyAF+yoenv+hBr1S+rwsUw) [(5)](https://paperpile.com/c/fzIBvJ/RUpHc). The pipeline included motion correction, co-registration of the median volume to the whole-head and brain-extracted inhomogeneity corrected T1w scan, and spatial smoothing with 6mm FWHM kernel. For assessing the amount of motion in the functional data the program *fsl_motion_outliers* was run on the raw functional data. FD-Jenkinson was calculated for each participant. ICA-AROMA was performed to clean noise and artifacts. Then the brain images were extracted and the inhomogeneity of the magnetic field was corrected. T1w files in native space were segmented, WM and CSF masks were thresholded at 99% of the robust-range, eroded once and transformed to EPI space using MultiLabel interpolation of ANTs [(6)](https://paperpile.com/c/fzIBvJ/76E1k) and the inverted BBR co-registration transformation. Mean WM/CSF was calculated and regressed out of the functional files with a glm. Finally, the files are transformed into FSL-MNI space at 2mm, using an MNI brain-mask. The data were assessed through a quality check. The criteria to pass the quality check included: (i) No realignment parameter >4mm/degree, (ii) Mean FD-Jenkinson <0.3, (iii) Spike-regression criterion: threshold every volumes' individual FD value at >0.25, if the amount of motion-affected volumes leads to <4min of motion-free data, the subject is excluded. Finally, for each subject, the time course of cortical and subcortical regions of the Harvard-Oxford atlas [(7)](https://paperpile.com/c/fzIBvJ/f7RVx) were exacted.

Group demographics are described, separately for each contrast-of-interest in Supplementary Table S1.

*Mddrest*

The *mddrest* dataset, collected by the REST-meta-MDD Project, is currently the largest database of MDD rs-fMRI, including 1,300 patients with MDD and 1,128 HC from 25 cohorts in China.

On average, each site contributed 52.0 ± 52.4 patients with MDD (range 13 to 282) and 45.1 ± 46.9 NCs (range 6 to 251). MDD patients had an average HAMD score of 20.5 ±4.5 (range 5.0 to 24.9). Most MDD patients were female (800 vs. 455 males) (19).

Each site participating in the data collection implemented a standardized preprocessing protocol on Data Processing Assistant for Resting-State fMRI (DPARSF) [(8)](https://paperpile.com/c/fzIBvJ/ntpDx). The consortium did not make available raw 4D fMRI data, but the time course for regions of interest in multiple atlases. Individual-level imaging processing was performed at each site using standardized DPARSF processing parameters. The preprocessing pipeline is well described in [(8,9)](https://paperpile.com/c/fzIBvJ/ntpDx+cHoKB) and consisted in (i) slice-timing correction, (ii) realignment by linear rigid transformation, (iii) co-registration to the mean functional image using a 6 degrees-of-freedom linear transformation without re-sampling, (iv) segmentation into gray matter, white matter and cerebrospinal fluid, (v) transformation from individual native space to MNI space were computed with the DARTEL tool [(8)](https://paperpile.com/c/fzIBvJ/ntpDx). To minimize head motion confounds, The Friston 24-parameter model [(10)](https://paperpile.com/c/fzIBvJ/XzjWC) was used to regress out head motion effects. Other sources of spurious variance like respiratory and cardiac effects were also removed from the data through linear regression using WM and CSF signals. Linear trends were included as a regressor to account for drifts in the blood oxygen level dependent (BOLD) signal. Temporal bandpass filtering (0.01-0.1Hz) was performed on all time series at the end.

The consortium provided ratings for quality inspection. Only participants with a quality score of three or higher were included in the study. The time course of cortical and subcortical regions of the Harvard-Oxford atlas [(7)](https://paperpile.com/c/fzIBvJ/f7RVx) were provided.

Group demographics are described, separately for each contrast-of-interest in Supplementary Table S1.

*Abide* preprocessing was done using the C-PAC pipeline (20) with the same parameters as in (21). The *Uk Biobank* was provided pre-processed in MNI format. The time courses of cortical and subcortical regions were similarly extracted using the Harvard-Oxford atlas.

Model Selection: Hyperparameters Search

For the linear and rbf SVM, within a 5-fold cross-validation scheme, hyper-parameters were optimized on the training set using another cross-validation — a nested cross-validation. The hyper-parameters chosen were the ones optimizing the prediction accuracy on the validation data. For linear-SVM we optimized the C parameter, and for rbf-SVM we optimized the C and gamma parameters. The C parameter trades off correct classification of training examples with maximization of the decision function’s margin. For larger values of C, a smaller margin will be accepted if the decision function is better at classifying all training points correctly. A lower C will encourage a larger margin, thus yielding a simpler decision function, at the cost of training accuracy. Therefore C behaves as a regularization parameter in the SVM. The gamma parameter defines how far the influence of a single training example reaches, with low values meaning ‘far’ and high values meaning ‘close’. Regularisation parameter C was distanced in powers of ten from 1 to 1000; gamma was searched between 0.0001 and 0.001. The linear-SVM search for the C parameter revealed that differences in C did not impact the accuracy of the results, therefore the default value of C = 1 was used. We used the machine learning python package Scikit-Learn^^[[1]](#footnote-0)^^ [(11,12)](https://paperpile.com/c/fzIBvJ/XNi96+JDkGT) to run the hyper-parameters search, and SVM classifiers.

Our GCN also underwent hyper-parameter tuning. We used a random search scheme for the optimization of numbers of layers in the model (**K**), dimension of the hidden layers (**H**), learning rate (α), batch size, and the dropout regularization parameter.

**K** allows learning graph embeddings based on K-order relationships between the nodes. It indicates the *depth* of the model, while **H** represents the feature dimension, the width of the model at the nodes in the hidden layers. “α” is an optimization parameter. It determines how quickly the model is adapted to the problem. A learning rate that is too large can cause the model to converge too quickly to a suboptimal solution, whereas a learning rate that is too small can cause the model to get stuck in a suboptimal minima. The *Batch size* refers to the number samples propagated through the network at each iteration. Dropout is a regularization technique specifically developed for neural networks: a percentage of units of a layer is temporarily removed at random from the network so that only a ‘thinned’ network is trained at a time. The percentage of units removed by dropout is the parameter that can be optimized. For a detailed explanation of related DL terminology please refer to [(13)](https://paperpile.com/c/fzIBvJ/eSGdS). For computational feasibility, GCN were tuned only on one of the cross-validation folds. The selected parameters were then applied to the other folds.

GCN architecture

The GCN consisted of two GCN layers, followed by an average pooling operation of each node’s features and concatenation in a single vector of all the resulting mean of the nodes’ features to reduce the dimensionality of the graph and to change the representation from a graph to a single feature vector for classification. Dropout was applied before classification, and the nonlinear *softmax* transformation was performed. Our GCN layers consisted of two steps. The first step aggregated using the arithmetical average of the representation vectors of all its immediate neighbors in the current layer, and the second step fed the concatenated vector to a fully connected layer with ReLU, the nonlinear activation function. The FC layers were optimized separately for each contrast.

Of note, the first step of the GCN-layer consists in averaging the features between ROIs (nodes) and their strong correlations (edges). Interhemispheric homologue brain regions tend to have strong FC and similar FC profile with the rest of the brain, which will be represented twice. If they contain highly important signals for classification, the signal-to-noise ratio of the average will increase in favor of the relevant signal. The prominent inter-hemispheric connections in the results (Figure3A) may reflect the nature of the way we constructed the GCN-layers.

Results: Statistical Significance

We assessed significance using permutation testing [(14)](https://paperpile.com/c/fzIBvJ/68MEh). The test statistic was compared against permutation values computed using the same method as the test statistic, under a random rearrangement of the labels of the dataset (A null hypothesis).

The permutation value (p-value) is defined as:

p-value$=\frac{1 + \sum_{n=1}^{N} I(y_{n}^{*}> x)}{N}$

Where $x$ is the test statistic, $y_{n}^{*}$ is the test static under a random permutation of the labels, $I()$ is the indicator function and $N$is the number of permutations [(15)](https://paperpile.com/c/fzIBvJ/c3Tzq).

We used *balanced accuracy* as our test statistic and ran the test for 1000 permutations to compute the p-value for all the reported experiments.

The threshold for statistical significance $\alpha$ was set at 0.05 and we further employed Bonferroni correction for multiple comparisons (15) by computing the threshold

$b$ = $\frac{\alpha}{m}$, where $m$ is the total number of experiments. The null hypothesis was rejected and a result considered statistically significant if the p-value$≼b$

Misclassification and sub-groups analyses

Exploration of when the classification went wrong could provide valuable information to understand the potential reasons why individuals there were not classified correctly and to gain knowledge about the influence of different technical and non-technical variables affecting the classification results. We focused on the main contrast of interest MDD vs HC, and reported results for the SVM-rbf classifier, the one that reaches the best average accuracy.

First we performed an univariate t-test on the functional connectivity matrices between the two groups (correctly classified and misclassified), then we explore if the two groups are differently distributed according to the subjects’ demographics and clinical characteristics such as sex, age and diagnosis, and according to technical variables of the data such as recoding site, scanner manufacturer and movement during scanning (average framewise displacement according to Jenkinson). These analyses should be interpreted in the light of the relative low classification accuracy of the classifier, and therefore are not conclusive but had the potential to shed light on reasons why the classification failed.

In either datasets, when performing a t-test on the FC of correctly classified subjects and the misclassified ones, none of the comparisons survived FDR correction for multiple testing (*psymri* t range from: -4.18 to 2.96 all p-corr>0.05, *mddrest* t range from : -4.18 to 2.88, all p-corr >0.05). Furthermore, we found no evidence for difference in any of the considered variables between correctly classified individuals and misclassified ones as evident from the descriptive presented in Figure S3.

In table S14 we reported the performance accuracy of the classifier for sub-groups of the test-set. Doing so, we hope to gain information regarding the contribution of these factors in the final accuracy. The analyses showed very similar accuracy between females and males (63.31 and 63 respectively), and between MDD and HC (62.5 and 63.9 respectively). The contribution of the different manufacturers showed some variability, with data collected with a GE MRI scanner obtaining the best accuracy (66.49) and while data collected by a Philips scanner obtained the lowest accuracy (53.51), this information probably reflecting the fact that the GE MRI scanner was the most represented in the training data. Performances clustered according to recording sites showed the highest variability, ranging from 47.6 to 87.45, once again confirming the adverse effect of data heterogeneity in performance.

SVM ablation study

We performed an ablation study to assess the importance of the regions of interest for the model SVM-rbf in classifying MDD vs HC in the *psymri* and *mddrest* dataset separately. We repeated the experiment for each cross-validation fold separately and calculated the mean drop in accuracy, as described in the main section. Table S7 shows the 10 highest mean accuracy drop and their standard deviation between folds for the two datasets.

Only the anterior division of the left Middle Temporal Gyrus resulted within the 10 regions showing the highest drop in accuracy (mean±SD over 5 folds: -1.61±0.58 for *psymri*, -1.22±1.2 for *mddrest*). As expected, the higher susceptibility of the results is reflected by standard deviation of the performance, visibly higher than the one in the GCN ablation experiments. Interestingly, even with the mentioned limitations, the ablations experiment on the *mddrest* dataset again identified the Thalami between the ten regions with most influence on the performance of the classifier.

These results are derived from classifiers with a relatively low accuracy and therefore, conclusions need to be taken with cautions.

Analyses using a functional atlas parcellation

In our main analyses, we calculated the functional connectivity using ROIs parcellation derived by the Harvard-Oxford atlas. The choice of the atlas was based on data availability and our own previous work [(16)](https://paperpile.com/c/fzIBvJ/4eFx). The Harvard-Oxford atlas is an anatomy based atlas, and might be sub-optimal to investigate and calculate functional connectivity matrices.

To assess if better performance would be achieved by using a functional atlas instead of the anatomical atlas Harvard-Oxford, we repeated the analyses of the main contrast of interest MDD vs HC using the brain parcellation provided by the Craddock atlas [(17)](https://paperpile.com/c/fzIBvJ/6J3t) on the best performing classifier, the SVM rbf on the *mddrest* dataset. For completeness, we repeated the analyses using the SVM linear and the GCN, as well. The analyses and evaluation of the results were performed exactly as described in the main text.

The performance, reported as balanced accuracy mean (standard deviation) over 5 folds are as follows: SVM-linear: 63.1(2.26), SMV-rbf: 63.8(0.01) and GCN 58.9(2.6).

These results are comparable with the one obtained by using the Harvard-Oxford atlas.

Classification using a fully connected neural network

In this study, we intended to exploit the specific capability of GCNs to work on the graph structure of FC data. To compare the GCN results with a model that does not use this knowledge, we repeated the analyses training a fully connected neural network on the main contrast of interest MDD vs HC. The model input was the flattened lower triangular matrix of the functional connectivity matrix and the performance was evaluated using 5-fold cross-validation as described in the main text.

Specifically, we used a fully connected neural network with 3 layers. The output dimension of each layer was 1024, 512, 2. These dimensions were selected based on a quick random search for the number of layers and the dimension of each layer on the validation set.

ReLu activations, and batch normalization were performed after each of the first 2 layers.

Before the last fully connected layer, the model had a dropout layer with a probability of 0.5 for regularization purposes.

The model was trained using an Adam optimizer with an initial learning of 0.001 and cosine annealing learning rate scheduler, for 200 epochs. The best model was selected based on the lowest loss in the validation set (subset of the training fold).

The model performed with a mean accuracy of 59.02 (3.16) on the *psymri* dataset, 61.08 (2.99) in the *mddrest* dataset and a mean accuracy of 59.8 (1.4) on the two datasets together.

The results are comparable to the ones obtained by the GCNs and the SVMs. This confirms that using GCN to exploit the graph-like structure of FC does not bring an advantage for the classification tasks.

Predicting symptom severity

The REST-meta-MDD consortium provided information regarding the HAM-D score (17 items version) for 1113 patients, and the PsyMRI consortium provided the same score for 333 patients. We used this information to build a model able to predict the score, hoping this would capture clinical variabilities within the patients group.

The analysis presents some limitations. Firstly, not all scholars would agree the test captures the complexity of a multi-symptoms disorder like MDD. Secondly, the sample of data available is small, probably too small for a task known to be more complex to solve than classification.

Being aware of these limitations and therefore of the low reliability of the results, we trained a GCN and a Support Vector Regression (SVR) with a rbf kernel, using the same parameters described in the main text and the same evaluation procedure, to predict the HAM-D score of MDD patients from the FC matrices. Results showed that neither model is able to predict HAM-D score from the FC matrices. The SVR-rbf obtained explained variance between 0.035 and 0.07, while the GCN output only the mean HAM-D of the training test. This is not an a-typical outcome for under-powered regression models and is an indication that the data are insufficient to learn the complex relation with HAM-D.

Statistical harmonization correction of sites difference

To address data heterogeneity resulting from acquisition via different scanners we have applied the combat harmonization algorithm [(18)](https://paperpile.com/c/fzIBvJ/ngIE)on the functional connectivity matrices. To avoid potential information leakage, we independently performed combat harmonization in the training set and applied the estimated model to the test set data. Combat harmonization was applied on the largest 10 sites of both dataset (all included sites N>50 in *mddrest*, N>30 in *psymri*) and the case-control differences were included as a covariate of interest to be protected.

Finally we trained the GCN on the harmonized residuals and reported the 5-fold test accuracies on both dataset for the contrast MDD vs HC in the table S15. The final results after combat harmonization is lower compared to no harmonization on the same subjects by 0.58 points and 4.12 points for the mddrest and psymri datasets respectively. To evaluate whether combat harmonization has successfully removed site effects we performed a multi-class classification task on the acquisition site of the largest 4 sites of the mddrest dataset pre- and post-combat. The results show that combat successfully removes site differences with site classification accuracies of 64.1% pre-combat and 29.8% post-combat. Our preliminary hypothesis is that given a large number of sites with relatively balanced class distribution and a unified pre-processing pipeline, the machine learning model optimized for the case-control task can learn site-invariant features which in turn diminishes the return of the harmonization techniques. This is also in line with recent work in the computer vision and medical domain [(19)](https://paperpile.com/c/fzIBvJ/lRj9) that show that current harmonization methods fail to achieve significant if any gains in out-of-distribution samples. Having said so, we argue this is an area that requires more in-depth investigation for functional connectivity data.

Finally, these results are not surprising given that the application of combat harmonization for functional correlations has failed to give any significant advantage in different cohorts in different works by our group when a code is used where there is no data leakage between the train and test datasets (https://github.com/Warvito/neurocombat_sklearn).

Reference

1. [Smith SM, Jenkinson M, Woolrich MW, Beckmann CF, Behrens TEJ, Johansen-Berg H, et al. Advances in functional and structural MR image analysis and implementation as FSL. Neuroimage. 2004;23 Suppl 1:S208–19.](http://paperpile.com/b/fzIBvJ/iSyAF)

2. [Smith S, Bannister PR, Beckmann C, Brady M, Clare S, Flitney D, et al. FSL: New tools for functional and structural brain image analysis [Internet]. Vol. 13, NeuroImage. 2001. p. 249. Available from:](http://paperpile.com/b/fzIBvJ/yoenv) <http://dx.doi.org/10.1016/s1053-8119(01)91592-7>

3. [Jenkinson M, Beckmann CF, Behrens TEJ, Woolrich MW, Smith SM. FSL [Internet]. Vol. 62, NeuroImage. 2012. p. 782–90. Available from:](http://paperpile.com/b/fzIBvJ/hBr1S) <http://dx.doi.org/10.1016/j.neuroimage.2011.09.015>

4. [Tustison NJ, Avants BB, Cook PA, Song G, Das S, van Strien N, et al. The ANTs cortical thickness processing pipeline [Internet]. Medical Imaging 2013: Biomedical Applications in Molecular, Structural, and Functional Imaging. 2013. Available from:](http://paperpile.com/b/fzIBvJ/rwsUw) <http://dx.doi.org/10.1117/12.2007128>

5. [Woolrich MW, Ripley BD, Brady M, Smith SM. Temporal autocorrelation in univariate linear modeling of FMRI data. Neuroimage. 2001 Dec;14(6):1370–86.](http://paperpile.com/b/fzIBvJ/RUpHc)

6. [Avants BB, Tustison NJ, Stauffer M, Song G, Wu B, Gee JC. The Insight ToolKit image registration framework. Front Neuroinform. 2014 Apr 28;8:44.](http://paperpile.com/b/fzIBvJ/76E1k)

7. [Makris N, Goldstein JM, Kennedy D, Hodge SM, Caviness VS, Faraone SV, et al. Decreased volume of left and total anterior insular lobule in schizophrenia. Schizophr Res. 2006 Apr;83(2-3):155–71.](http://paperpile.com/b/fzIBvJ/f7RVx)

8. [Chao-Gan Y, Yu-Feng Z. DPARSF: A MATLAB Toolbox for “Pipeline” Data Analysis of Resting-State fMRI. Front Syst Neurosci. 2010 May 14;4:13.](http://paperpile.com/b/fzIBvJ/ntpDx)

9. [Yan C-G, Chen X, Li L, Castellanos FX, Bai T-J, Bo Q-J, et al. Reduced default mode network functional connectivity in patients with recurrent major depressive disorder. Proc Natl Acad Sci U S A. 2019 Apr 30;116(18):9078–83.](http://paperpile.com/b/fzIBvJ/cHoKB)

10. [Friston KJ, Williams S, Howard R, Frackowiak RS, Turner R. Movement-related effects in fMRI time-series. Magn Reson Med. 1996 Mar;35(3):346–55.](http://paperpile.com/b/fzIBvJ/XzjWC)

11. [Avila J, Hauck T. scikit-learn Cookbook: Over 80 recipes for machine learning in Python with scikit-learn. Packt Publishing Ltd; 2017. 374 p.](http://paperpile.com/b/fzIBvJ/XNi96)

12. [Garreta R, Moncecchi G. Learning scikit-learn: Machine Learning in Python. Packt Publishing Ltd; 2013. 100 p.](http://paperpile.com/b/fzIBvJ/JDkGT)

13. [LeCun Y, Bengio Y, Hinton G. Deep learning. Nature. 2015 May 28;521(7553):436–44.](http://paperpile.com/b/fzIBvJ/eSGdS)

14. [Edgington E, Onghena P. Randomization Tests, Fourth Edition. CRC Press; 2007. 376 p.](http://paperpile.com/b/fzIBvJ/68MEh)

15. [Bonferroni CE. Teoria statistica delle classi e calcolo delle probabilità. 1936. 62 p.](http://paperpile.com/b/fzIBvJ/c3Tzq)

16. [El Gazzar A, Cerliani L, van Wingen G, Thomas RM. Simple 1-D Convolutional Networks for Resting-State fMRI Based Classification in Autism. In: 2019 International Joint Conference on Neural Networks (IJCNN). 2019. p. 1–6.](http://paperpile.com/b/fzIBvJ/4eFx)

17. [Craddock RC, James GA, Holtzheimer PE 3rd, Hu XP, Mayberg HS. A whole brain fMRI atlas generated via spatially constrained spectral clustering. Hum Brain Mapp. 2012 Aug;33(8):1914–28.](http://paperpile.com/b/fzIBvJ/6J3t)

18. [Yu M, Linn KA, Cook PA, Phillips ML, McInnis M, Fava M, et al. Statistical harmonization corrects site effects in functional connectivity measurements from multi-site fMRI data. Hum Brain Mapp. 2018 Nov;39(11):4213–27.](http://paperpile.com/b/fzIBvJ/ngIE)

19. [Zhang H, Dullerud N, Seyyed-Kalantari L, Morris Q, Joshi S, Ghassemi M. An empirical framework for domain generalization in clinical settings. In: Proceedings of the Conference on Health, Inference, and Learning. New York, NY, USA: Association for Computing Machinery; 2021. p. 279–90. (CHIL ’21).](http://paperpile.com/b/fzIBvJ/lRj9)

20. Craddock, C., Sikka, S., Cheung, B., Khanuja, R., Ghosh, S.S., Yan, C., Li, Q., Lurie, D., Vogelstein, J., Burns, R. and Colcombe, S., 2013. Towards automated analysis of connectomes: The configurable pipeline for the analysis of connectomes (c-pac). *Front Neuroinform*, *42*, pp.10-3389.

21. Craddock, C., Benhajali, Y., Chu, C., Chouinard, F., Evans, A., Jakab, A., Khundrakpam, B.S., Lewis, J.D., Li, Q., Milham, M. and Yan, C., 2013. The neuro bureau preprocessing initiative: open sharing of preprocessed neuroimaging data and derivatives. *Frontiers in Neuroinformatics*, *7*, p.27.

Supplementary Figures


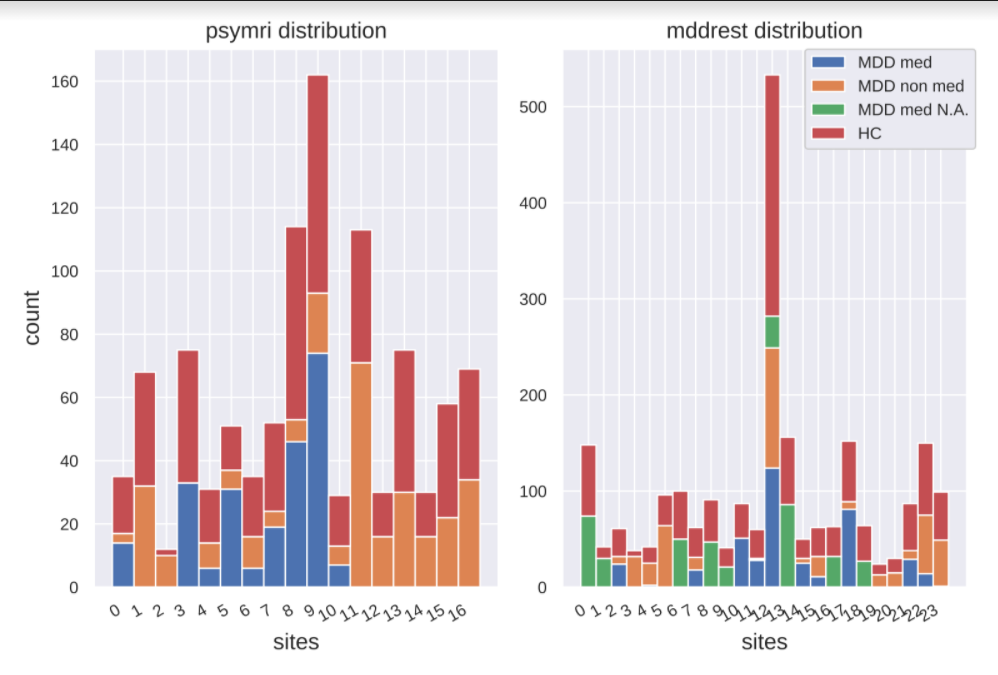


Figure S1. Sample distribution for each site in the *psymri* and *mddrest* datasets, indicating the number of medicated MDD patients (MDD med), non-medicated MDD patients (MDD non-med), MDD patients whose medication status is not available (MDD med N.A.) and the number of healthy controls (HC)


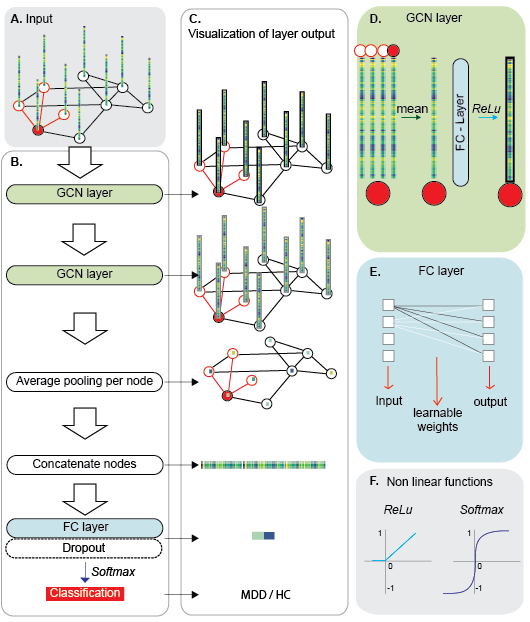


Figure S2:. Illustrative representation of GCN and related concepts. A. Input of the GCN is a graph representing the FC of a single subject. In the graph representation, each node is an ROI, the node feature is the correlational profile between the node ROI and the other ROIs in the brain. The ROIs characterized by strong correlation are connected by edges. B. Schematic representation of our GCN model. It consists of two GCN layers, where spatial aggregation and non-linear transformation are applied to the node signals to arrive at higher level graph feature representations. Graph features are then averaged and flattened to a 1-D vector representing the N nodes of the graph. Finally the graph vector representation is passed on to a fully connected layer with dropout for classification. C. Visualization of the output of each layer of the model to show the major transformation happening to the input. A layer and visual representation of the output are connected by a black arrow. D. Schematic representation of the operations taking place in a GCN layer for an example node. First, the features of a node and its neighbours are aggregated and averaged. Next, the result vector is input to a FC layer. Finally the signal is transformed by the non-linear function ReLu. E. Schematic representation of a FC layer, in which all neurons in the input


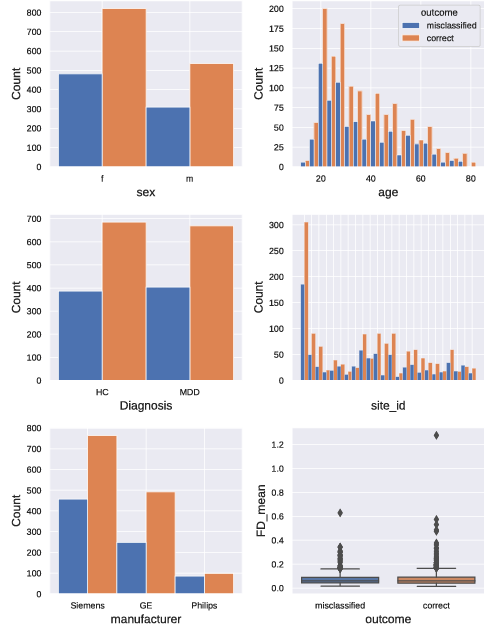


Figure S3. Distribution of misclassified and correctly classified individuals according to sex, age, diagnosis, recoding site, scanner manufacturer and movement during scanning (average framewise displacement according to Jenkinson) for the contrast MDD vs HC, *mddrest* dataset, SVM-rbf.

Supplementary tables

| **contrast** | **dataset** | **group** | **n sites** | **n** | **male/female/n.a.** | **age mean(sd)** |
| --- | --- | --- | --- | --- | --- | --- |
| MDD vs HC | | | | | | |
|  | psymri | MDD | 13 | 275 | 166/109/0 | 33.7 (11.6) |
|  | psymri | HC | 13 | 275 | 179/96/0 | 35.1 (12.2) |
|  | mddrest | MDD | 24 | 974 | 562/411/1 | 36.6 (15.7) |
|  | mddrest | HC | 24 | 974 | 603/371/0 | 36.1 (14.7) |
| MDD non med vs HC | | | | | | |
|  | psymri | MDD non-med | 7 | 62 | 35/27/0 | 37.4 (12.7) |
|  | psymri | HC | 7 | 62 | 34/28/0 | 36.2 (11.6) |
|  | mddrest | MDD non-med | 15 | 255 | 153/101/1 | 35.9 (15.1) |
|  | mddrest | HC | 15 | 255 | 154/101/0 | 33.9 (13.3) |
| MDD med vs HC | | | | | | |
|  | psymri | MDD med | 7 | 106 | 61/45/0 | 32.2 (10.6) |
|  | psymri | HC | 7 | 106 | 72/34/0 | 35.2 (13.2) |
|  | mddrest | MDD med | 12 | 217 | 128/89/0 | 41.5 (17.8) |
|  | mddrest | HC | 12 | 217 | 139/78/0 | 42.0 (15.3) |
| MDD med vs MDD nonmed | | | | | | |
|  | psymri | MDD med | 7 | 35 | 17/18/0 | 37.9 (12.0) |
|  | psymri | MDD non-med | 7 | 35 | 21/14/0 | 37.5 (12.7) |
|  | mddrest | MDD med | 11 | 183 | 121/62/0 | 36.9 (14.2) |
|  | mddrest | MDD non-med | 11 | 183 | 113/70/0 | 38.6 (13.8) |
| male vs female | | | | | | |
|  | psymri | male | 10 | 215 | 215/0/0 | 36.4 (13.0) |
|  | psymri | female | 10 | 215 | 0/215/0 | 33.6 (11.0) |
|  | mddrest | male | 24 | 797 | 797/0/0 | 36.8 (15.3) |
|  | mddrest | female | 24 | 797 | 0/797/0 | 35.2 (15.4) |

Table S1. Demographic information about the sample for each comparison. Note that equal group sizes were enforced for each comparison by random selection of participants for the smallest group. MDD: major depressive disorder, med: medicated, non-med: non-medicated, HC: healthy controls, sd: standard deviation.

Table S2. Classification performance for each classifier, comparison, and dataset. Results are represented as mean balanced accuracy (standard deviation) across five folds. Results marked with ‘*’ are significantly different from chance. P-values are corrected for multiple comparisons using Bonferroni correction. SVM: support vector machine, GCN: graph convolution network, MDD: major depression disorder, med: medicated, non-med: non-medicated, HC: healthy controls

Table S3. F-score, sensitivity and specificity results of each classifier and contrast, *psymri* dataset. The mean performance of different methods in each contrast with 5-fold cross validation (standard deviation shown within brackets). SVM: support vector machine, GCN: graph convolution network, f1: f-score, sens: sensitivity, spec: specificity, MDD: major depression disorder, med: medicated, non-med: non-medicated, HC: healthy controls

Table S4. F-score, sensitivity and specificity results of each classifier and contrast, *mddrest* dataset. The mean performance of different methods in each contrast with 5-fold cross validation (standard deviation between brackets). SVM: support vector machine, GCN: graph convolution network, f1: f-score, sens: sensitivity, spec: specificity, MDD: major depression disorder, med: medicated, non-med: non-medicated, HC: healthy controls

Table S5. F-score, sensitivity and specificity results of each classifier and contrast, *psymri+mddrest* dataset. The mean performance of different methods in each contrast with 5-fold cross validation (standard deviation between brackets). SVM: support vector machine, GCN: graph convolution network, f1: f-score, sens: sensitivity, spec: specificity, MDD: major depression disorder, med: medicated, non-med: non-medicated, HC: healthy controls

Table S6. GCN ablation study results for the HC vs MDD contrast on the *mddrest* dataset, the table shows the top-10 regions with the highest mean test accuracy drop when masking out in the test set

Table S7. SVM rbf ablation study results for the MDD vs HC contrast on the *mddrest* dataset, the table shows the top-10 regions with the highest mean test accuracy drop when masking out in the test set

Table S8. T-test results for the contrast MDD vs HC, *psymri* dataset. Correlations significantly different between MDD patients and HC, in the *psymri* dataset. T values and p-values, FDR corrected, are reported. R: Right, L: Left

Table S9. T-test results for the contrast MDD vs HC, *mddrest* dataset. Correlations significantly different between MDD patients and HC, in the *mddrest* dataset. T values and p-values, FDR corrected, are reported. R: Right, L: Left

Table S10. T-test results for the contrast MDD non-med vs HC, *psymri* dataset. Correlations significantly different between MDD non-med patients and HC, in the *psymri* dataset. T values and p-values, FDR corrected, are reported. R: Right, L: Left

Table S11. T-test results for the contrast MDD med vs HC, *psymri* dataset. Correlations significantly different between MDD med patients and HC, in the *psymri* dataset. T values and p-values, FDR corrected, are reported. R: Right, L: Left

Table S12. T-test results for the contrast MDD med vs HC, *mddrest* dataset. Correlations significantly different between MDD med patients and HC, in the *mddrest* dataset. T values and p-values, FDR corrected, are reported. R: Right, L: Left

Table S13. T-test results for the contrast MDD med vs MDD non-med, *psymri* dataset. Correlations significantly different between MDD med and non-med patients, in the *psymri* dataset. T values and p-values, FDR corrected, are reported. R: Right, L: Left

|  |  | tot n test set | accuracy % |
| --- | --- | --- | --- |
| sex | | | |
|  | f | 1300 | 63 |
|  | m | 845 | 63,31 |
| diagnosis | | | |
|  | HC | 1072 | 63,9 |
|  | MDD | 1073 | 62,35 |
| manufacturer |  |  |  |
|  | GE | 740 | 66,49 |
|  | Philips | 185 | 53,51 |
|  | Siemens | 1220 | 62,54 |
| recoding site | | | |
|  | S1 | 139 | 64,75 |
|  | S2 | 51 | 47,06 |
|  | S3 | 58 | 53,45 |
|  | S5 | 21 | 66,67 |
|  | S6 | 28 | 60,71 |
|  | S7 | 81 | 69,14 |
|  | S8 | 141 | 63,83 |
|  | S9 | 91 | 71,43 |
|  | S10 | 37 | 62,16 |
|  | S11 | 58 | 74,14 |
|  | S12 | 34 | 52,94 |
|  | S13 | 36 | 55,56 |
|  | S14 | 93 | 63,44 |
|  | S15 | 89 | 66,29 |
|  | S16 | 58 | 67,24 |
|  | S17 | 85 | 49,41 |
|  | S18 | 35 | 48,57 |
|  | S19 | 81 | 87,65 |
|  | S20 | 490 | 62,24 |
|  | S21 | 147 | 60,54 |
|  | S22 | 44 | 72,73 |
|  | S23 | 55 | 47,27 |
|  | S24 | 54 | 62,96 |
|  | S25 | 139 | 64,75 |

Table S14. Performance of the classifier SVM-rbf on the test-set, contrast MDD vs HC, *mddrest* dataset, divided per sub-groups of interest

| **Dataset** | **without combat** | **With combat** |
| --- | --- | --- |
| mddrest | 61.24 (1.79) | 60.66 (2.1) |
| psymri | 60.58 (2.77) | 56.46 (1.71) |

Table S15. Mean accuracy results (standard deviation) with and without combat harmonization for the contrast MDD vs HC

1. <https://scikit-learn.org/> [↑](#footnote-ref-0)
